# Comparison of 2.5D and 3D Quantification of Femoral Head Coverage in Normal Control Subjects and Patients with Hip Dysplasia

**DOI:** 10.1371/journal.pone.0143498

**Published:** 2015-11-24

**Authors:** Hui Cheng, Li Liu, Weimin Yu, Hong Zhang, Dianzhong Luo, Guoyan Zheng

**Affiliations:** 1 Division of Joint Surgery, Dept. Orthopaedic Surgery, The First Affiliated Hospital of PLA General Hospital, Beijing, China; 2 Institute for Surgical Technology and Biomechanics, University of Bern, CH-3014, Bern, Switzerland; University of Brescia, ITALY

## Abstract

Hip dysplasia is characterized by insufficient femoral head coverage (FHC). Quantification of FHC is of importance as the underlying goal of the surgery to treat hip dysplasia is to restore a normal acetabular morphology and thereby to improve FHC. Unlike a pure 2D X-ray radiograph-based measurement method or a pure 3D CT-based measurement method, previously we presented a 2.5D method to quantify FHC from a single anteriorposterior (AP) pelvic radiograph. In this study, we first quantified and compared 3D FHC between a normal control group and a patient group using a CT-based measurement method. Taking the CT-based 3D measurements of FHC as the gold standard, we further quantified the bias, precision and correlation between the 2.5D measurements and the 3D measurements on both the control group and the patient group. Based on digitally reconstructed radiographs (DRRs), we investigated the influence of the pelvic tilt on the 2.5D measurements of FHC. The intraclass correlation coefficients (ICCs) for absolute agreement was used to quantify interobserver reliability and intraobserver reproducibility of the 2.5D measurement technique. The Pearson correlation coefficient, r, was used to determine the strength of the linear association between the 2.5D and the 3D measurements. Student’s t-test was used to determine whether the differences between different measurements were statistically significant. Our experimental results demonstrated that both the interobserver reliability and the intraobserver reproducibility of the 2.5D measurement technique were very good (ICCs > 0.8). Regression analysis indicated that the correlation was very strong between the 2.5D and the 3D measurements (r = 0.89, p < 0.001). Student’s t-test showed that there were no statistically significant differences between the 2.5D and the 3D measurements of FHC on the patient group (p > 0.05). The results of this study provided convincing evidence demonstrating the validity of the 2.5D measurements of FHC from a single AP pelvic radiograph and proved that it could serve as a surrogate for 3D CT-based measurements. Thus it may be possible to use this method to avoid a CT scan for the purpose of estimating 3D FHC in diagnosis and post-operative treatment evaluation of patients with hip dysplasia.

## Introduction

Hip dysplasia is characterized by insufficient femoral head coverage (FHC) [1]. Due to smaller weight-bearing surface and increased contact stresses, hip dysplasia has been implicated as a main cause of hip osteoarthritis (OA) [2, 3]. Growing evidence supports the theory that its severity increases the chance of OA [4]. One of the most important goals of the surgical treatment of patients with hip dysplasia is to improve FHC and thereby to restore a normal acetabular morphology [5]. Thus, improved FHC is one of the strong predictor of the treatment outcome [6].

Two-dimensional (2D) anteriorposterior (AP) pelvic radiograph is the standard imaging means that is widely used in evaluating dysplastic hips. Although it has an inferior accuracy in comparison to three-dimensional (3D) techniques based on Computed Tomography (CT), it is used routinely because of its simplicity, availability, and minimal expense associated with its acquisition. In literature, different standard geometric parameters were presented to quantify hip dysplasia, such as extrusion index [7], lateral center-edge angle (LCE) [8], anterior center edge angle (ACE) [9], and others [10]. Though easy to measure from an AP pelvic X-ray radiograph, all these parameters are indirect indicators for assessing FHC and their accurate interpretations are subjected to substantial errors if the individual pelvis orientation with respect to X-ray plate is not taken into consideration.

Apart from classic measurement techniques with a goniometer, computer programs were introduced as useful and reproducible tools for interpretation of AP pelvic radiographs. Pedersen et al. [11] reported a computer-assisted measurement program for the analysis of radiographic parameters of hip dysplasia, which for each hip used 16 landmarks extracted from an AP pelvic radiograph to computer 12 morphological parameters. Another method introduced by Engesæter et al. [12], which used a program to estimate the radiological measurements by manually defining 46 different radiological landmarks. More recently, Nepple et al. [13] reported a computer-assisted measurement program (HipMorphometry; Orthopaedic Research Institute, USA) to perform radiographic analysis. All these previously introduced methods [11–13] quantify the morphology of dysplastic hips with pure 2D measurements.

Alternatively, subject-specific 3D reconstructions of pelvis and femur morphology, generated from a volumetric CT data, have been used as a more accurate way of quantifying 3D coverage of the femoral head. Klaue et al. [14] proposed a CT evaluation method estimating coverage and congruency of hip joint. They made topographical map of the acetabulum and the femoral head from the cross section images of a CT scan and calculated the FHC. More recently, Dandachli et al. [15] described a new CT-based evaluation method for dysplastic hips with the assumption that femoral head is simplified to be an ideal sphere. Previously, we developed a 3D CT-based measurement technique to quantify FHC [16], where automatically detected acetabular rims were used to differentiate the covered and uncovered areas of the femoral head. Although CT-based methods have a higher accuracy in comparison with the 2D radiography-based techniques, CT is only acquired in rare cases with severe pelvic deformations and exposes the patient to higher radiation.

Unlike the pure 2D X-ray radiograph-based methods or the pure 3D CT-based measurement methods, previously we presented a program to conduct 2.5D quantification of various hip morphological parameters from a single AP pelvic X-ray radiograph (Hip^2^Norm [17, 18]; University of Bern, Bern, Switzerland). This program is able to correct the projected acetabular rims and the corresponding radiographic hip parameters caused by pelvic malorientation based on a cone-beam projection model. In addition, this program allows for calculating FHC not only from the AP image but also from a simulated craniocaudal image. Recently, this program has been validated to be a reliable and accurate tool to estimate FHC from a plain X-ray radiograph in patients with femoroacetabular impingement (FAI) [19]. However, the precise relationship between 2.5D and 3D measurements of FHC in patients with hip dysplasia has never been investigated. Furthermore, the influence of pelvic tilt during X-ray image acquisition on the accuracy of the 2.5D measurements of the FHC is unknown.

In this study, we are aiming to answer following questions:

1. Is there significant difference in 3D measurements of FHC between the normal control subjects and the patients with hip dysplasia?
2. Is the 2.5D measurement method a reliable and reproducible tool for quantification of FHC in the normal control subjects and the patients with hip dysplasia?
3. How does the pelvic tilt during X-ray image acquisition affect the 2.5D measurement of FHC of the patients with hip dysplasia?
4. Is there significant difference in 2.5D measurements of FHC between the normal control subjects and the patients with hip dysplasia?

In order to answer these questions, the 2.5D measurements will be calculated with the Hip^2^Norm software on Digitally Reconstructed Radiographs (DRRs). By using DRRs instead of real plain radiographs, we are able to precisely control the pelvis orientation during image acquisition in a simulated environment, thereby to investigate the influence of pelvic tilt on the accuracy of the 2.5D measurements. For all experiments, 3D CT-based measurements of FHC will be taken as the gold standard.

## Materials and Methods

The study cohort contained 2 groups, i.e., the patient group and the control subject group. The usage of the data of the patient group was approved by Medical Ethics Committee, The First Affiliated Hospital of PLA General Hospital, Beijing, China and the usage of the data of the control subject group was approved by Medical Ethics Committee, The First Affiliated Hospital of Zhejiang Chinese Medical University, Hangzhou, China. Patient records/information of both groups were anonymized and de-identified prior to analysis. The control group (42 hips) consist of subjects without morphological hip abnormalities and the patient group (40 hips) consist of patients with hip dysplasia. All control subjects had no complains which were related to hip or pelvis diseases. Control hips were excluded if the patients can be diagnosed as hip dysplasia or FAI radiographically. All patients in the latter group were diagnosed clinically as having hip dysplasia (LCE angle < 20° [8] and regional symptom). Hips in patient group were excluded, if important anatomical landmarks, such as anterior superior iliac spine (ASIS) and tear drops, were missing in CT data.

### 2.5D Measurements of Femoral Head Coverage on DRRs

#### DRRs

**Fig 1:**
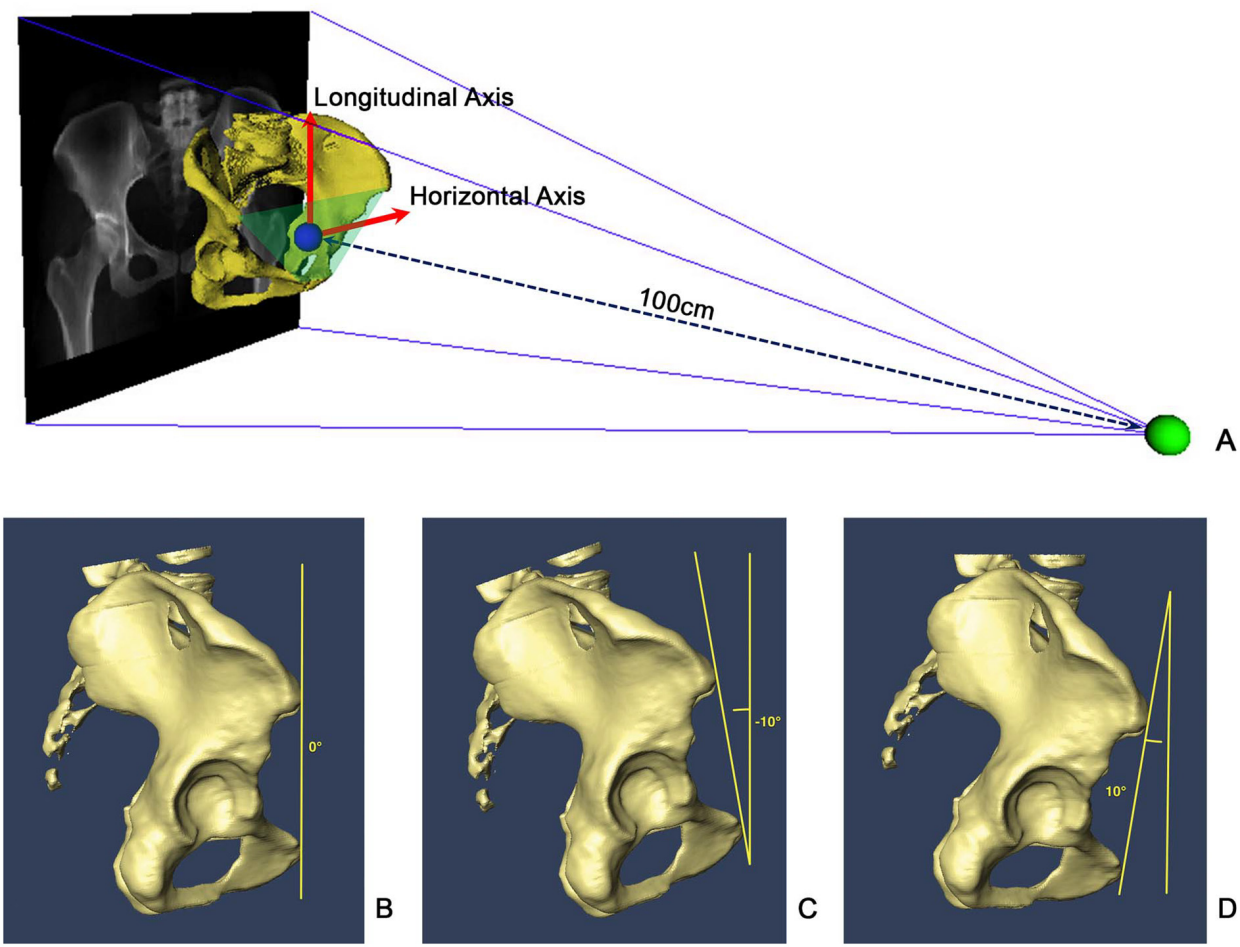
DRRs with different amounts of pelvic tilt. (A) Schematic view of DRR by controlling the projection parameters. (B) AP view (0° of pelvic tilt). (C) T-10 oblique view (-10° of pelvic tilt). (D) T+10 oblique view (10° of pelvic tilt).

DRR is a ray-casting technique to simulate projections from user-defined perspectives by quantitatively controlling pelvic posture, as well as x-ray projection parameters such as source-film distance, X-ray source, and field of view. The anterior pelvic plane (APP) was used as a reference plane to control the pelvic posture, which is established by anatomical landmarks consisting of the bilateral ASISs and the bilateral pubic tubercles (PTs). More specifically, the longitudinal axis of the pelvis was defined by connecting the middle points of bilateral ASISs and PTs. The horizontal axis of pelvis was defined to be parallel to the line connecting bilateral ASISs. In this study, we set the source-to-APP distance as 100cm (Fig 1(A)).

Initially, APP was set to be parallel with the virtual X-ray table and the longitudinal axis of the pelvis was upwards to produce an image similar to a clinical AP radiograph, which means 0° of pelvic rotation and tilt. The pelvic tilt was then changed by rotating the APP around the horizontal axis of the pelvis with an decremental of 10° starting from 10° until -30° to get 5 DRRs for each patient. We defined anterior pelvic tilt as positive, posterior pelvic tilt as negative. These five projections were selected to simulate AP, 10° pelvic tilt view (T+10), -10° pelvic tilt view (T-10), -20° pelvic tilt view (T-20) and -30° pelvic tilt view (T-30), respectively (see Fig 1(B), 1(C) and 1(D) for examples).

#### 2.5D measurements of Femoral Head Coverage

Hip^2^Norm was used to generate 2.5D measurements from all DRRs. This program allows for a computerized evaluation of the most commonly used radiographic hip parameters from a single AP pelvic X-ray radiograph or DRR.

**Fig 2:**
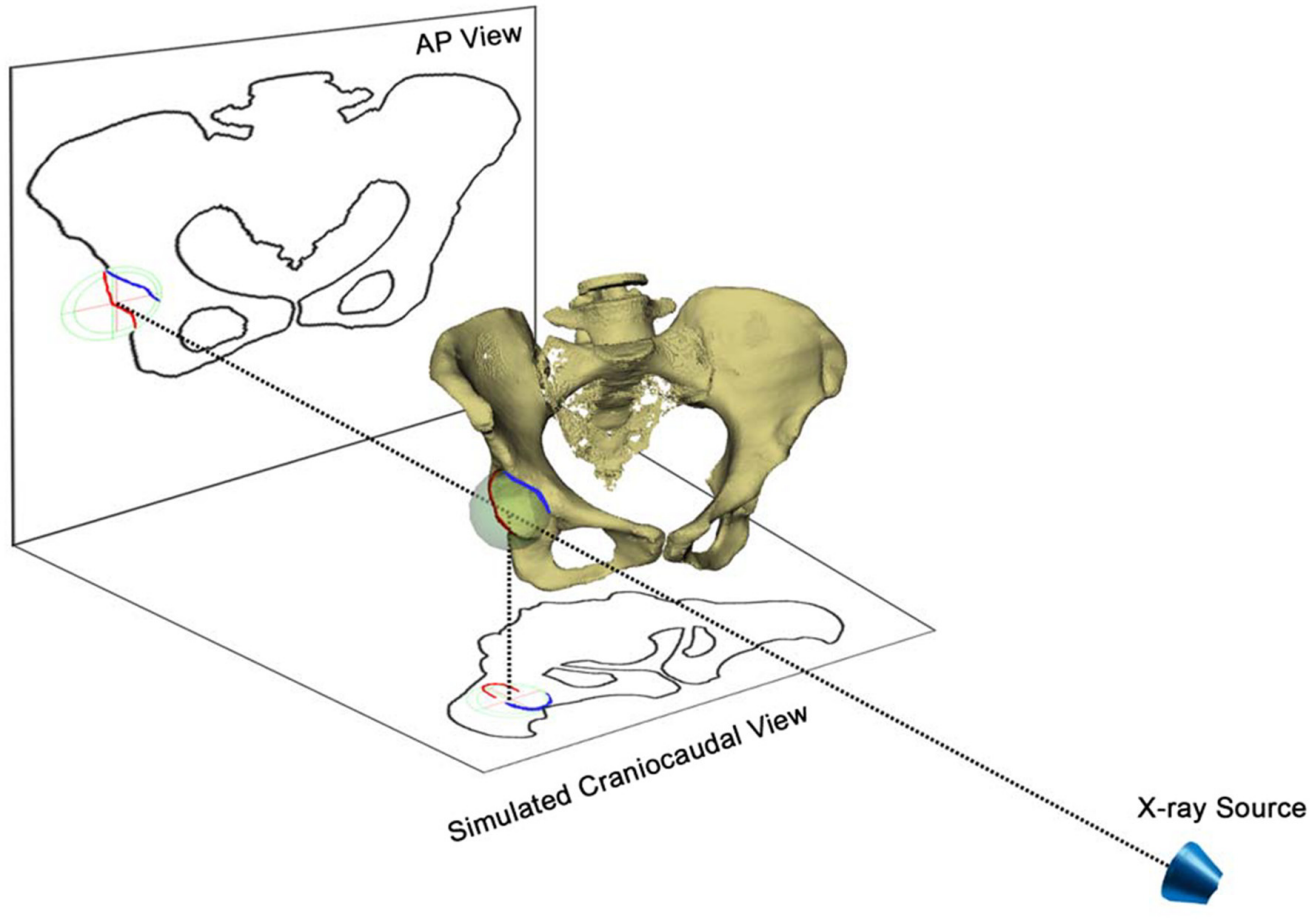
A schematic view of reconstructing 3D hip joints from an AP pelvic radiograph and a cone-beam projection model. Individual pelvic tilt/rotation can be corrected with the help of the vertical/horizontal distances between the symphysis and the sacrococcygeal joint. The craniocaudal view is a simulated head-to-foot parallel projection and is used to calculate the FHC in the simulated craniocaudal image.

Hip^2^Norm belongs to the shadow-casting methods [20]. These methods are based on a model where the radiographic shadows of pelvic landmarks are casted back toward the point source of the beam to determine the relative position of the objects (Fig 2). The virtual 3D hip joints are reconstructed from an AP radiograph using a cone-beam projection model. Such a 3D reconstruction allows for generation of a simulated 2D craniocaudal image, as shown in Fig 2.

**Fig 3:**
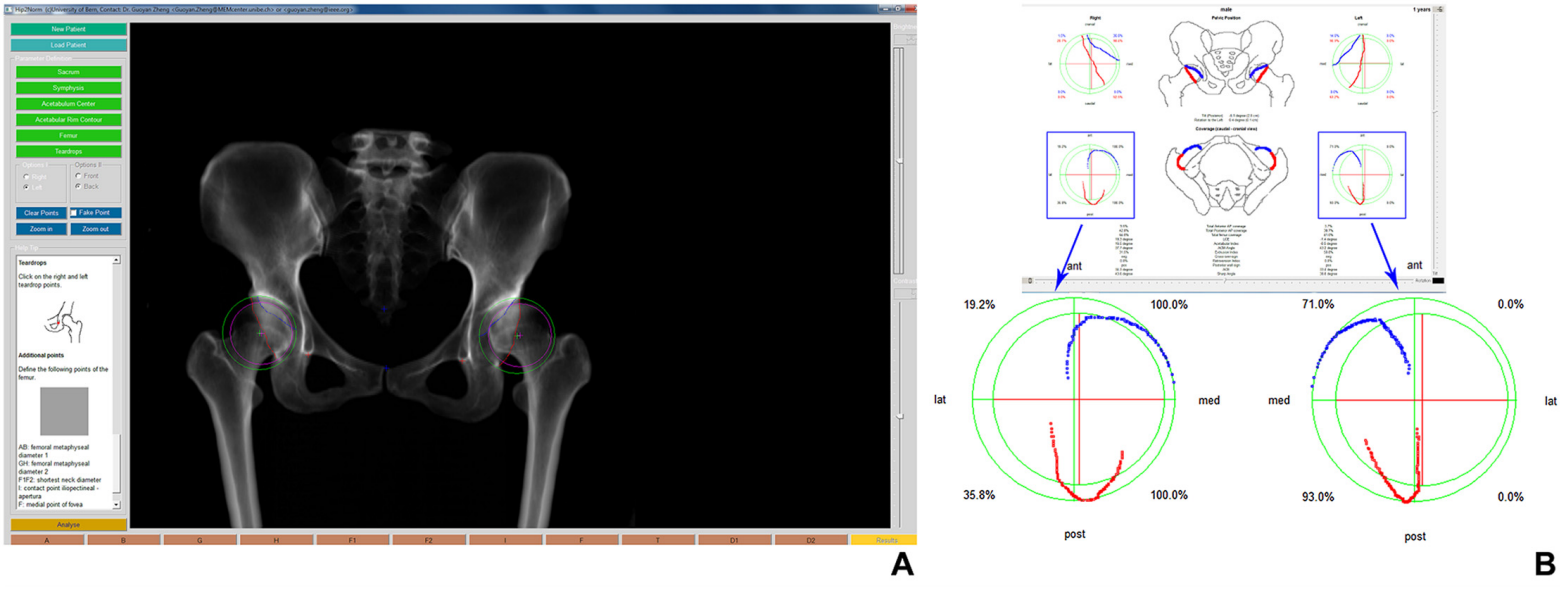
Hip^2^Norm Software. (A) Graphical user interface (left image) for digitizing landmarks for computerized evaluation of an AP radiograph of the pelvis. The inferior margins of the teardrops (red crosses), the outline of the projected anterior and posterior acetabular rim (blue and red line), and the middle of the sacrococcygeal joint (upper blue cross) and the upper border of the symphysis (lower blue cross) must be drawn manually, while the center and the radius of femoral head (pink cross) and acetabulum (green cross) are obtained by fitting a circle to three points specified by the user. (B) Anterior and posterior acetabular rims, and 2.5D measurements of FHC after neutralizing the pelvic position (right image).

In addition, Hip^2^Norm allows for a correction of the rotation and tilt of the pelvis during image acquisition to a neutral position. For details about how Hip^2^Norm corrects the rotation, obliqueness and tilt of the pelvis in an acquired X-ray radiograph, we refer interested readers to our previous work [17, 18]. After correction, a set of standard hip morphological parameters can be calculated by Hip^2^Norm including total and partial FHC in the AP X-ray radiograph as well as in the simulated craniocaudal image [14], LCE angle [8], etc. (see Fig 3 for an example). In this study the primary focus is on the craniocaudal FHC [14] calculated with Hip^2^Norm [17, 18].

### 3D CT-based Measurements of Femoral Head Coverage

The CT-based 3D FHC measurement technique [21] used in this study is adapted from our previous method reported in [16]. The difference is that our current method is based on native geometry of the femoral head. In contrast, our previous work assumed that the femoral head is ideally spherical [16]. In normal hips the assumption is valid since the femoral head is spherical or nearly so. However, in a dysplastic hip, the femoral head may be elliptical or deformed [22]. Thus the method used in this study is more accurate than the method that we introduced in [16].

Here a 3D measurement of FHC is defined to be a ratio between the area of the upper femoral head surface covered by the acetabulum and the area of the complete upper femoral head surface from the weight-bearing point of view [23]. This is because although the entire articular surface of the acetabulum is involved in weight-bearing, the resultant joint force of the hip at the stance phase of gait comes mainly from the superior or anterosuperior direction as reported in [23]. Therefore, the superior hemisphere of the femoral head and the opposing acetabular surface are major weight-bearing areas. The 3D CT-based measurement is then calculated with following algorithm. The inputs to this algorithm are femoral surface model, acetabular rim points [16] and the axial plane which is perpendicular to the APP and passes through the femoral head center.

**Fig 4:**
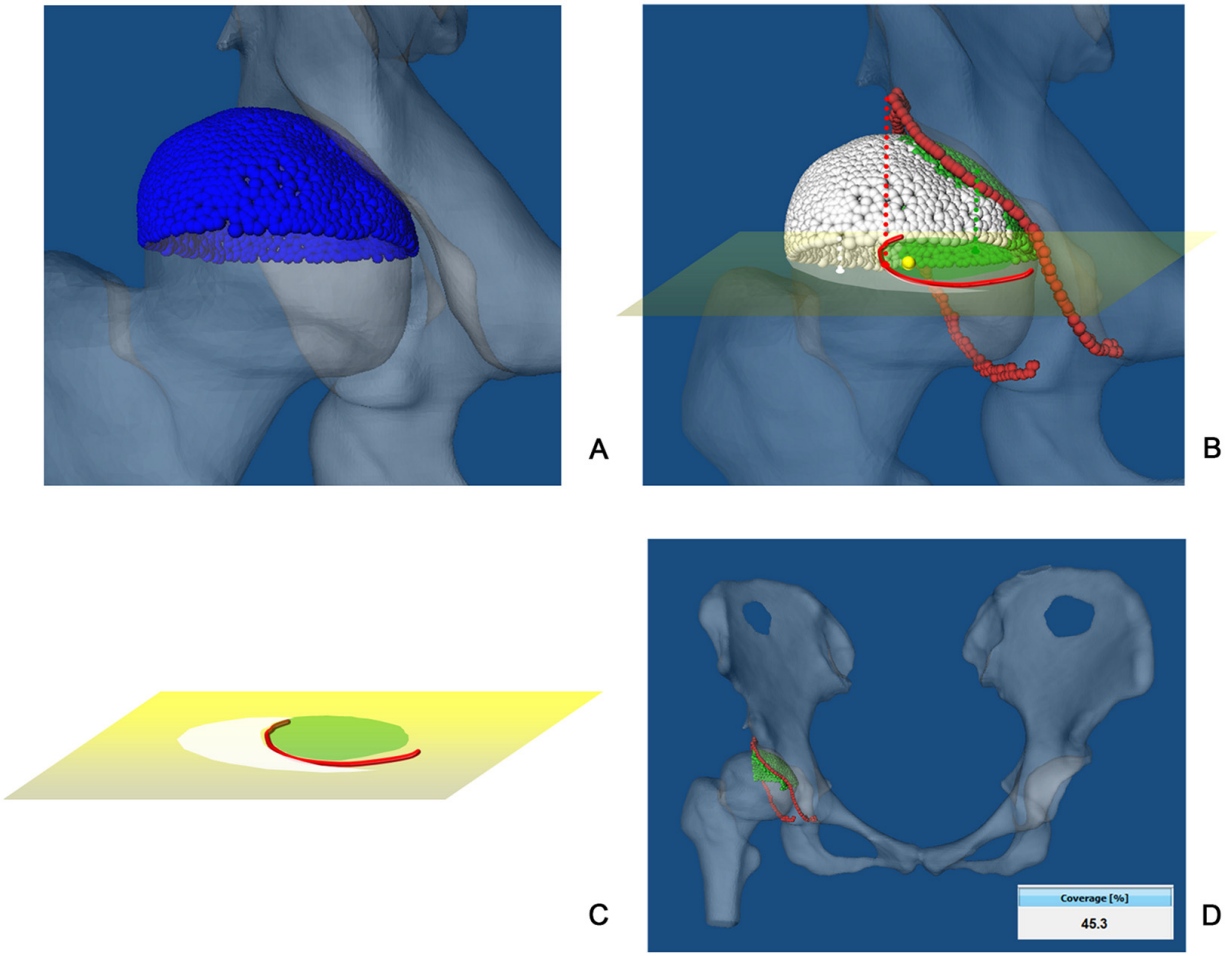
3D measurement of FHC. (A) The superior surface of the native femoral head (approximated with blue triangle meshes) and the opposing acetabular surface are major weight-bearing areas. (B) Cranial rim points of the acetabulum and the superior hemisphere are projected onto the axial plane to produce a circle and a curved line (the projected acetabular rim contour) cutting across it. (C) A topographical image on the axial plane represents the femoral head with its covered (green area) and uncovered (white area) parts. (D) The percentage of FHC is calculated as a ratio between the green area and the sum of the green and the white areas on the femoral head.

**Step 1:** Only the superior weight-bearing surface of the femoral head is used to estimate coverage as shown in Fig 4(A). The cranial rim points of the acetabulum and the superior hemisphere are both projected on to the axial plane to produce a circle and a curved line (the projected acetabular rim contour) cutting across it (Fig 4(B)).

**Step 2:** A topographical image is generated on the axial plane which represents the total femoral head. The covered and uncovered areas are separated by the projected acetabular rim contour. The green and white areas represent covered and uncovered parts, respectively (Fig 4(C)).

**Step 3:** The percentage of FHC is calculated as a ratio between the area of covered part and the area of the total femoral head (see Fig 4(D) for details).

### Study design

Our study was divided into five experiments:

1. The first experiment was designed to compare 3D measurements of the FHC between the patient group and the control group.
2. The second experiment was designed to investigate the interobserver reliability and the intraobserver reproducibility of the 2.5D measurement technique.
3. The third experiment was designed to quantify bias, precision and correlation between the 2.5D measurements and the 3D measurements of the FHC on both the control group and the patient group.
4. The fourth experiment was designed to investigate the effect of pelvic tilt on 2.5D measurements of the FHC on the patient group taking 3D measurements as the gold standard.
5. The fifth experiment was designed to compare the 2.5D measurements of the FHC between the patient group and the control group.

### Statistical analysis

Statistical analysis was performed using SPSS (V19.0; IBM Corp., Armonk NY). Kolmogorov-Smirnov test was used to determine data normality.

Both the interobserver reliability and the intraobserver reproducibility of the 2.5D measurement technique were quantified using the intraclass correlation coefficient (ICC) for absolute agreement, with 95% confidence intervals (CI). 2.5D measurements from each read and from each observer were averaged to a single value. Interobserver reliability was evaluated using the averaged values. Agreement was interpreted as: poor if the ICC < 0.20, fair if 0.21–0.40, moderate if 0.41–0.60, good if 0.61–0.80 and very good if > 0.80 [24].

Linear regression was used to determine the correlation between the 2.5D and the 3D measurements on both the control group and the patient group. For linear regression analysis, independent variable was defined as the 2.5D measurements of FHC. Dependent variable was defined as the 3D CT-based measurements of FHC. The strength of the correlation was assessed using the Pearson correlation coefficient, r, which was interpreted as follows: very weak if r = 0–0.19, weak if 0.20–0.39, moderate if 0.40–0.59, strong if 0.60–0.79 and very strong if 0.80–1.00 [25].

Bias was defined as the average difference between the 2.5D measurements and the 3D measurements. Precision was defined as one standard deviation of difference including 68% of comparison points [19]. Student’s t-test was used to determine whether there were significant differences between different measurements on different groups. The significant level was set to as *α* = 0.05.

## Results

**Fig 5:**
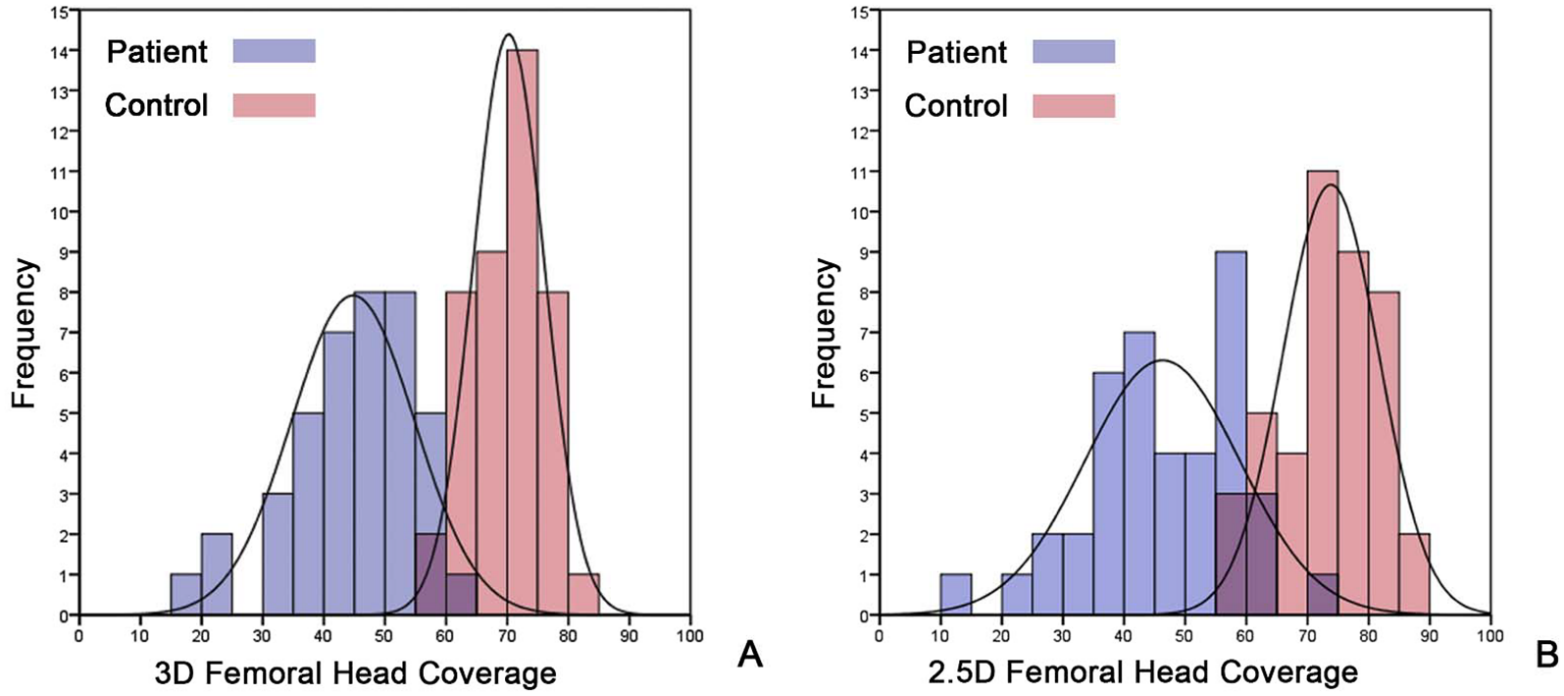
(A) Distributions of 3D FHC measurements on two study groups. (B) Distributions of 2.5D FHC measurements on two study groups.

In the first experiment we found that the 3D measurements of FHC varied from 57.9% to 80.2% on the normal control subjects. The mean FHC was 70.1% (SD 5.7%). In contrast, FHC on the patient group varied from 17.9% to 60.8%. The mean FHC was 44.7% (SD 10.1%). With Kolmogorov-Smirnov test, the 3D measurements of FHC on both the control and the patient groups were determined as normally distributed (p > 0.75). We then fit two normal curves to the 3D measurement distributions, one for the measurements on the control group and the other for the patient group, as shown in Fig 5(A). Student’s t-test showed that there were statistically significant differences between the FHC measured on the control group and that measured on the patient group (p < 0.001). As shown in Fig 5(A), the fitted normal curve for each group has a mode, and the overlap part of the measurements from these two study groups were very small (3 hips). The intersection point between the two fitted normal curves is around 60% FHC, which can be taken as the threshold to differentiate the patient group from the control group (Fig 5(A)).

In the second experiment, as shown in Table 1, both the interobserver reliability and the intraobserver reproducibility of the 2.5D measurement technique were found to be very good (ICCs > 0.8).

**Table 1:** Intra- and interobserver reproducibility of the 2.5D measurement technique.

	Intraobserver 1	Intraobserver 2	Interobserver
Control	0.81 (0.65–0.90)	0.88 (0.78–0.94)	0.88 (0.76–0.94)
Patient	0.96 (0.92–0.98)	0.97 (0.94–0.98)	0.97 (0.94–0.98)

**Fig 6:**
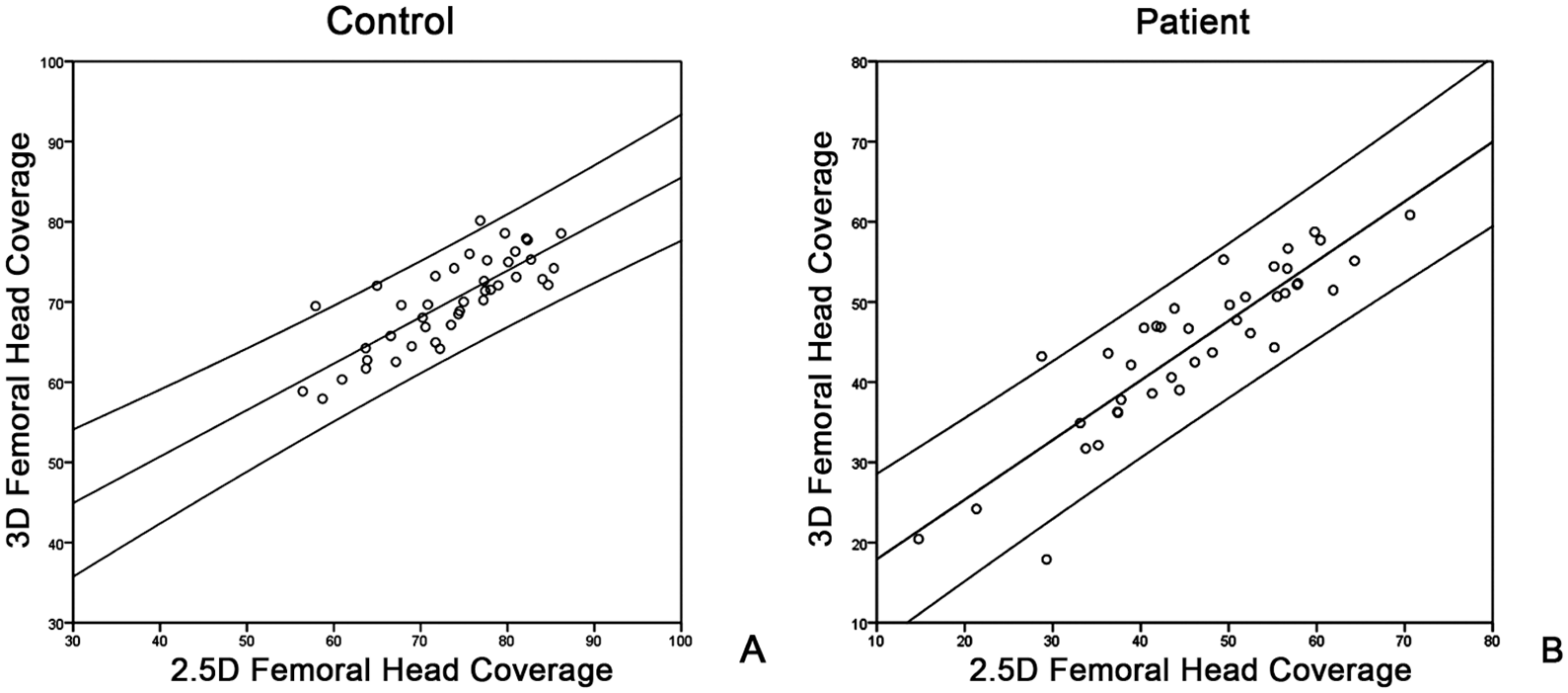
(A) Scatter plot of the 2.5D measurements of the FHC against the 3D measurements on control group. (B) Scatter plot of the 2.5D measurements of the FHC against 3D measurements on patient group.

In the third experiment we found that the average 2.5D measurement of the FHC was slightly larger than the average 3D measurement on the control group. The bias measured on the control group was about 4.6% of the average 3D measurement (Table 2). Regression analysis showed that the correlation was very strong between the 2.5D and the 3D measurements of FHC (r = 0.80, p < 0.001) on the control group (Fig 6(A)). For the group of patients with hip dysplasia, the bias was 2.9% of the associated average 3D measurement (Table 2). Regression analysis indicated a very strong correlation between the 2.5D measurements and the 3D measurements (r = 0.89, p < 0.001) of FHC on the patient group (Fig 6(B)) as well. Student’s t-test showed that there were no statistically significant differences between the 2.5D and the 3D measurements of FHC on the patient group (p = 0.14).

**Table 2:** Bias, precision and Pearson correlation coefficient between the 2.5D and the 3D measurements of FHC on both the control and the patient groups.

Groups	Bias	Precision	r
Control	3.4	4.7	0.80
Patient	1.3	5.6	0.89

**Fig 7:**
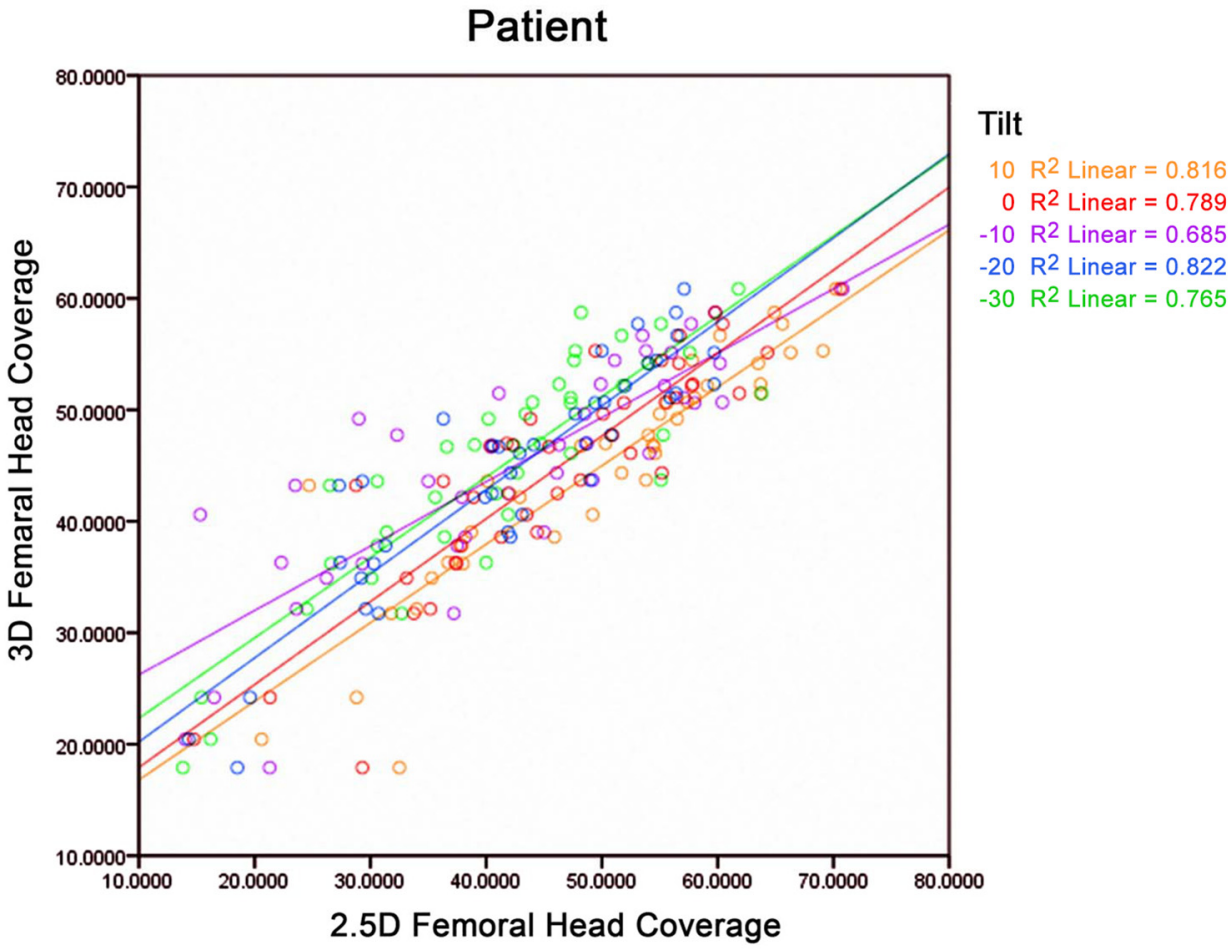
Scatter plot of 2.5D measurements of the FHC from different views against 3D measurements of the FHC on patient group.

In the fourth experiment, the correlations between the 2.5D measurements of the FHC using DRRs generated from different oblique views (T+10, T-10, T-20, T-30) and the 3D measurements were very strong (r > 0.8) (see Fig 7 and Table 3 for details). The biases and precisions of the 2.5D measurements from these oblique views (T+10, T-10, T-20, T-30) slightly increased in comparison with those measured from the AP view, but the strengths of the correlation were all very strong (Fig 7).

**Table 3:** Bias, precision and Pearson correlation coefficient between 3D measurements of FHC and 2.5D measurements of FHC on the patient group using different oblique views.

Tilt	Bias	Precision	r
10°	4.9	5.8	0.90
-10°	-2.7	8.3	0.83
-20°	-2.1	5.2	0.94
-30°	-2.5	9.6	0.87

In the fifth experiment we found that the 2.5D measurements of FHC varied from 56.4% to 86.2% on the control group. The mean FHC was 73.5% (SD 7.9%). In contrast, FHC measured on the patient group varied from 14.8% to 70.6%. The mean FHC was 46.1% (SD 12.0%). Kolmogorov-Smirnov test demonstrated that the 2.5D measurements of the FHC on both the control group and the patient group are normally distributed (p > 0.80). Again we respectively fit two normal curves to the 2.5D measurement distributions, one for the measurements on the control group and the other for the patient group, as shown in Fig 5(B). It was found that the overlap part for the 2.5D measurements on these two study groups was slightly larger than those with the 3D measurements (7 hips vs. 3 hips). We also observed statistically significant differences between control subjects and patients with hip dysplasia (p < 0.001). For the 2.5D measurements, the intersection point between the two fitted normal curves is around 62% FHC, which means that one can use this threshold to differentiate the patient group from the control group (Fig 5(B)).

## Discussion

Deficient FHC of a dysplastic hip has been recognized as a significant precursor of osteoarthritis [26]. There existed different methods to measure the FHC of a dyplastic hip. Both 3D CT-based and 2D X-ray radiograph-based methods have been introduced before.

The first purpose of this study was to quantify and compare 3D FHC between the normal control group and the patient group. We determined that patients with hip dysplasia had a statistically significant smaller FHC than subjects in the control group. The mean FHC for the patients with hip dysplasia was found to be 44.7% while the mean FHC for the subjects in the control group was found to be 70.1%. Our findings were consistent with the results reported in [15], where using a CT-based method, they reported a mean FHC of 73% for patients with dysplastic hips and a mean FHC of 51% for subjects in the control group. Although both studies are based on 3D CT data, the methods used to quantify the FHC are different. More specifically, their method assumed a spherical shaped femoral head while in our study we used a method that is based on the native shape of the femoral head. Their assumption may be valid for subjects in the control group but for patients with dyplastic hips, such an assumption is questionable due to the fact that the femoral head of a dyspastic hip may be elliptical or deformed [22]. This probably explained why we have obtained slightly different mean FHCs from theirs. Furthermore, by fitting two normal curves to the 3D measurements of the two study groups respectively, we determined a threshold of 60% FHC to differentiate a patient with hip dysplasia from a normal subject. The clinical relevance of this finding may have important implications for the diagnosis and treatment of patients with dysplastic hips. More specifically, the 3D FHC threshold can be used as a more accurate criterion to diagnose hip dysplasia than 2D criteria that are established on other surrogate parameters such as LCE angle.

3D CT-based measurement techniques, though accurate, as demonstrated in this study and other studies [14, 15], are not widely used in clinical routine due to high radiation dosage caused by the CT acquisition. In comparison with the acquisition of a single AP X-ray radiograph, a CT acquisition may induce as high as 150–200 times radiation dose to a patient [27]. Repeated use of CT scans for preoperative and postoperative hip morphology quantification will also significantly increase the radiation exposure, leading to higher cancer risk. [28–30]. Due to this limitation, surgeons usually depend on surrogate parameters such as LCE angle measured on 2D X-ray radiographs to quantify hip morphology.

This is the reason why we started to investigate the 2.5D measurement technique for quantifying FHC from a plain X-ray radiograph. Different from previous attempts [23], our 2.5D measurement technique can automatically correct pelvic malorientation. In this study, we investigated the interobserver reliability and the intraobserver reproducibility of our 2.5D measurement technique. Moreover, taking the 3D measurements as the gold standard, we quantified the biases, precisions and Pearson correlation coefficients between the 2.5D and the 3D measurements on both the patient group and the control group. It was found that both intraobserver reliability and interobserver reproducibility were very good. The study result showed that there was not only strong correlation but also small bias between the 2.5D and the 3D measurements, even in patients with previous surgery history.

In order to investigate the influence of the pelvic tilts on the 2.5D measurements of FHC, for each patient we performed the 2.5D measurements on 5 DRRs with different pelvic tilts. Taking the 3D measurements as the gold standard, we compared the 2.5D measurements on these 5 DRRs with 3D measurements. By using DRRs we are able to precisely control the pelvis orientation in a simulated environment, thereby to investigate the influence of the pelvic tilt on the accuracy of the 2.5D measurements. In clinical routine, X-ray radiographs are not always acquired in a standardized way. The pelvic tilt range [-30°, 10°] as used in this study was chosen based on values reported in literature. It was reported in [31] that the angles of pelvic tilt range from +3° to -17° in the lying position and from +3° to -27° in the standing posture. We found very strong correlations between the 3D measurements and the 2.5D measurements in each pelvic tilt positions. The results of this study demonstrated that due to the fact that our 2.5D measurement technique can automatically correct the pelvic malorienation, the influence of pelvic tilts on the accuracy of our 2.5D measurement technique is small.

We finally quantified the 2.5D measurement distributions on both the control group and the patient group. We found that the distribution profiles of the 2.5D measurements were quite similar to those of the 3D measurements. The intersection point between the fitted normal curves of the 2.5D FHC measurements was around 62%. The clinical relevance of this finding may have important implications for the diagnosis and treatment of patients with dysplastic hips when CT acquisition is not allowed. More specifically, when only 2D X-ray radiograph is available, the 2.5D FHC threshold can be used as an accurate criterion to diagnose hip dysplasia or to evaluate treatment efficacy.

It is worth to mention the limitations of this study. Although our 2.5D measurement technique has the advantage to automatically correct pelvic malorientaiton, it is still based on the assumption of a spherical hip joint. In this study, we only evaluated the overall accuracy of the 2.5D measurements taking the associated 3D CT-based measurements as the gold standard. We did not quantify the influence of the severity of the femoral head deformity on the accuracy of our 2.5D measurement technique. Nonetheless, our experimental results demonstrated that even for patients with hip dysplasia, the 2.5D measurement technique can be applied to get a reasonably accurate quantification of FHC.

In summary, in this paper we first quantified and compared 3D FHC between the normal control group and the patient group. We found that a threshold of 3D FHC could be used to differentiate subjects in the control group from the patients with hip dysplasia. Taking the 3D measurements as the gold standard, we then conducted comprehensive experiments to investigate various aspects of our 2.5D FHC measurement technique. Based on the morphological features extracted from 2D X-ray radiograph, our 2.5D measurement technique first reconstructs a 3D hip joint model, and then projects the 3D model onto a simulated craniocaudal view in order to compute FHC. It is neither a pure 2D measurement, nor a true 3D measurement. Therefore, we regarded it as a 2.5D measurement technique. As demonstrated by the results in our study, the 2.5D FHC measurement technique can be used to avoid a CT scan for the purpose of estimating FHC in diagnosis and post-operative treatment evaluation of patients with hip dysplasia.
